# Intercalated chemotherapy and erlotinib for advanced NSCLC: high proportion of complete remissions and prolonged progression-free survival among patients with EGFR activating mutations

**DOI:** 10.2478/raon-2014-0038

**Published:** 2014-11-05

**Authors:** Matjaz Zwitter, Karmen Stanic, Mirjana Rajer, Izidor Kern, Martina Vrankar, Natalija Edelbaher, Viljem Kovac

**Affiliations:** 1 Institute of Oncology Ljubljana, Ljubljana, Slovenia; 2 Faculty of Medicine, University of Maribor, Maribor, Slovenia; 3 University Hospital for Pulmonary Diseases Golnik, Golnik, Slovenia; 4 Department of Pulmonary Medicine, University Clinical Centre Maribor, Maribor, Slovenia

**Keywords:** Non-small cell lung cancer, EGFR activating mutations, gemcitabine, cisplatin, erlotinib, intercalated therapy, metabolic response

## Abstract

**Background:**

Pharmaco-dynamic separation of cytotoxic and targeted drugs might avoid their mutual antagonistic effect in the treatment of advanced non-small cell lung cancer (NSCLC).

**Patients and methods.:**

Eligible patients were treatment-naive with stage IIIB or IV NSCLC. In addition, inclusion was limited to never-smokers or light smokers or, after 2010, to patients with activating epidermal growth-factor receptor (EGFR) mutations. Treatment started with 3-weekly cycles of gemcitabine and cisplatin on days 1, 2 and 4 and erlotinib on days 5 to 15. After 4 to 6 cycles, patients continued with erlotinib maintenance.

**Results:**

Fifty-three patients were recruited into the trial: 24 prior to 2010 (of whom 9 were later found to be positive for EGFR mutations), and 29 EGFR mutation-positive patients recruited later. Unfavourable prognostic factors included stage IV disease (51 patients - 96%), performance status 2–3 (11 patients - 21%) and brain metastases (15 patients -28%). Grade 4 toxicity included 2 cases of neutropenia and 4 thrombo-embolic events. The 15 EGFR negative patients had 33% objective response rate, median progression-free survival (PFS) 6.0 months and median survival 7.6 months. Among 38 EGFR positive patients, complete response (CR) or partial response (PR) were seen in 16 (42.1%) and 17 (44.7%) cases, respectively. PET-CT scanning was performed in 30 patients and confirmed CR and PR in 16 (53.3%) and 9 (30.0%) cases, respectively. Median PFS for EGFR mutated patients was 21.2 months and median survival was 32.5 months.

**Conclusions:**

While patients with EGFR negative tumors do not benefit from addition of erlotinib, the intercalated schedule appears most promising for those with EGFR activating mutations.

## Introduction

Discovery of activating epidermal growth-factor receptor (EGFR) mutations as strong predictors of response to targeted therapy with tyrosine-kinase inhibitors (TKIs) has dramatically changed the therapeutic options for a subset of patients with non-small cell lung cancer (NSCLC). Several randomised trials of first-line treatment have confirmed superiority of TKIs gefitinib, erlotinib or afatinib over platinum-based doublets^1^–^3^ and led to registration of these drugs for first-line treatment of metastatic EGFR-mutated NSCLC.

After a median interval of 9 to 14 months, the majority of EGFR positive patients treated with TKIs experience a relapse. While cytotoxic drugs or a different TKI may lead to a second remission, long-term prognosis is unfavourable.

To date, there have been few successful attempts to prevent or delay the development of resistance to TKIs and to extend time to progression.^4^ Four large randomized trials on non-selected population of patients with all histologic types showed overlapping curves of progression-free survival (PFS) and overall survival (OS) with chemotherapy and concomitant TKI, as compared to chemotherapy alone.^5^–^8^ Due to this negative experience, few researchers believe that further attempts to combine the two classes of drugs are justified.

To address the issue of the optimal schedule for combination of chemotherapy with TKIs, we should understand why simultaneous therapy with both classes of drugs failed. An explanation may be in the fact that TKIs cause cell cycle arrest and accumulation of cells in G1, leading to their lesser sensitivity to cytotoxic drugs.^9^,^10^ Mutual antagonistic effect of cytotoxic drugs and gefitinib has been confirmed on lung cancer cell lines harbouring sensitizing EGFR mutations.^11^

Pharmacodynamic separation of chemotherapy and of targeted drugs has been proposed for their synergistic activity. Observations on NSCLC cell lines showed that the sequence of cytotoxic drugs and TKIs is crucial for optimal result.^12^ Compared to single-agent docetaxel, docetaxel followed by erlotinib resulted in significantly enhanced apoptosis. However, in the reverse sequence of erlotinib followed by docetaxel, a reduction of apoptosis was observed. An interval of 6 days without erlotinib was found to be sufficient to allow cells to re-enter the cycle and to restore their sensitivity to chemotherapy.^13^

Here we present experience from a Phase II clinical trial of intercalated therapy with chemotherapy and erlotinib for treatment-naive patients with advanced adenocarcinoma of the lung. The trial started in 2005 when testing for EGFR mutations was not yet available. To enrich the proportion of patients with tumors sensitive to erlotinib, the initial protocol limited inclusion to never-smokers or light smokers. In 2009, testing for EGFR became available. Analysis of archived biopsy samples for the initial cohort of 24 patients revealed a clear and statistically significant difference in response, PFS and OS in favour of EGFR positive patients. While EGFR wild type patients had response rate of 30% and median time to progression of 6 months, all patients with EGFR activating mutations responded to treatment, with 21.5 months as median time to progression.^14^ An amendment to the protocol was therefore made and all additional patients had to be positive for activating mutations of EGFR.

The primary objectives of the trial were progression-free survival and response to treatment; secondary endpoints were treatment toxicity and overall survival. At the time of amendment, metabolic response was added as an additional secondary objective.

## Patients and methods

### Eligibility criteria

Patients eligible for the trial were chemo-naive with non-squamous lung carcinoma; had stage III B unsuitable for chemo-radiotherapy with curative intent or stage IV; had measurable disease; and had adequate parameters of hematological, liver, renal and cardiac function to receive platinum-based chemotherapy. Patients with asymptomatic untreated brain metastases, and patients in stable neurological status after treatment for brain metastases with surgery and/or radiotherapy were eligible.

In addition to the above criteria, the initial protocol limited inclusion to never-smokers or light smokers with a history of less than 10 pack-years. An amendment made in September 2010 replaced this limitation by confirmed activating mutations of EGFR.

All patients were fully informed and provided written consent to participate in the trial.

### Initial diagnostics

Within four weeks prior to treatment, the extent of the disease was determined by chest X-ray and CT scanning of the chest, upper abdomen and brain. Since 2010, PET-CT scanning was included in the initial diagnostics and in evaluation of response to treatment.

EGFR status was assessed by EGFR mutation analysis. To test for EGFR mutations, genomic DNA was extracted from formalin-fixed, paraffin-embedded tumor tissue sections. Quantification of extracted DNA was done on Qubit Fluorometer (Invitrogen, Carlsbad, USA). To detect EGFR gene activating mutations, the first 10 patients were tested with TheraScreen EGFR29 Mutation Kit (DxS Diagnostics, Qiagen, Manchester, UK) and afterwards withCobas 4800 (Roche Molecular Systems, Pleasanton, USA).

### Treatment

The treatment started with four to six cycles of intercalated chemotherapy and erlotinib according to the following schedule:

**Table:** 

Day 1:	gemcitabine 1250 mg/m^2^
Day 2:	cisplatin 75 mg/m^2^, with appropriate hydration and antiemetics
Day 4:	gemcitabine 1250 mg/m^2^
Days 5–15:	erlotinib 150 mg daily
The cycle was repeated on day 22.	

Standard criteria for dose reduction, delay or omission of cytotoxic drugs were observed. Cisplatin was replaced by carboplatin at AUC 5 in case of grade 2–3 nausea or vomiting or in case of grade 1 nephrotoxicity. The intercalated phase of treatment was terminated for patients with any grade 4 hematological toxicity, grade ≥2 nephrotoxicity or any other grade ≥3 non-hematological toxicity, in which case the treatment would continue with maintenance erlotinib.

Immediately after the last cycle, patients continued with maintenance erlotinib 150 mg daily until progression or unacceptable toxicity. In case of grade ≥2 skin toxicity, local antibiotics and/or vitamin K1 cream were applied^15^ and reduction of the dose of erlotinib was considered.

### Response, time of progression, and follow-up

Definitions of complete response (CR), partial response (PR), stable disease (SD) and progression followed the RECIST criteria.^16^ The first evaluation was done during the third cycle, with confirmation of response during the 5th cycle.

Metabolic response to treatment was an additional secondary endpoint. PET-CT scanning was performed prior to treatment and repeated at 6 months after commencing the treatment. Control PET examination included all initial sites of disease, with measurement of corresponding maximal standard uptake value (SUV). Appearance of any new lesion or increase in SUV of a previously known lesion together with ≥ 20% increase in its size was declared as progression. For partial remission, all previously known lesions should either disappear or show at least a 50% reduction of uptake. Patients between progression and partial response were classified as stable disease. Finally, normalisation of PET-CT and disappearance of all lesions with initially increased SUV was required to declare a CR.^17^,^18^

### Statistical planning

In the initial study protocol, the sample size was calculated on the basis of expected median PFS of 10 months with the intercalated schedule, to be compared with 6 months as PFS for the combination of gemcitabine and cisplatin. Planning for the sample size was reviewed in 2010 when TKIs became the new standard first-line treatment for EGFR mutated patients. Taking 20 months as the expected PFS for the intercalated regimen, 35 patients with EGFR mutations were needed for a 80% power to confirm, at the one-sided 0.10 significance level, a difference to the reported 12 months as median PFS for monotherapy with erlotinib.^19^

### Ethical considerations

The investigators strictly followed the Helsinki Declaration and the European Council Convention on Protection of Human Rights in Bio-Medicine (Oviedo 1997). The protocol was approved by the Institutional Review Board Committee (Institute of Oncology, Ljubljana) and by the National Committee for Medical Ethics. The trial was registered with the European Medicines Agency, EudraCT Number: 2010-023362-44.

## Results

The first cohort of 24 patients selected on the basis of histologic type and smoking history was recruited between September 2005 and July 2010. Among these patients, 9 were later found to be positive for EGFR mutations. After that date and until October 2013, additional 29 patients with EGFR activating mutations entered the trial.

The series includes 28 women and 25 men. All patients were Caucasians. While the majority of patients were in fair general condition, 8 patients had performance status (PS) 2 and additional 3 patients had PS 3. Two patients had stage IIIB unsuitable for treatment with radiotherapy with curative intent; all other patients had stage IV disease. Demographics, sites of metastatic disease and types of EGFR mutations are presented in Table 1.

### Treatment delivery and acute toxicity

The actual number of cycles of intercalated therapy was from 1 to 6 (median: 4 cycles).

During the induction phase of the treatment, 6 patients had grade 4 toxicity: two had grade 4 neutropenia and 4 developed deep vein thrombosis, in 3 cases followed by pulmonary embolisms. These 6 patients continued treatment with TKI maintenance. Due to grade 2 – 3 nausea, vomiting or asthenia, additional 4 patients received only 3 cycles of intercalated therapy and continued with monotherapy with erlotinib. During the maintenance phase of the treatment, the only serious and common side effect was skin toxicity, with grades 2 and 3 in 16 and 14 patients, respectively (Table 2).

### Response to treatment, progression-free survival, second-line treatment and survival

All patients were evaluable for response and no patient has been lost to follow-up. Due to significant differences between EGFR wild-type and mutated disease, these two groups of patients will be presented separately.

#### EGFR wild-type or unknown (15 patients)

Among patients with EGFR wild-type tumors, 5 patients had PR, 8 had minimal response or stable disease and 2 had progression. Remissions were short-lived with median PFS 6.0 months (95% confidence interval [CI] 3.9 – 8.1). The most frequent sites of progression were intrathoracic disease (11 patients), bone (5) or brain (3). Eight patients did not receive further systemic treatment; other options were continuation with erlotinib (5 patients) or chemotherapy (2). Median survival was 7.6 months (95% CI 5.0 – 10.2).

#### EGFR activating mutations (38 patients)

**FIGURE 1.**
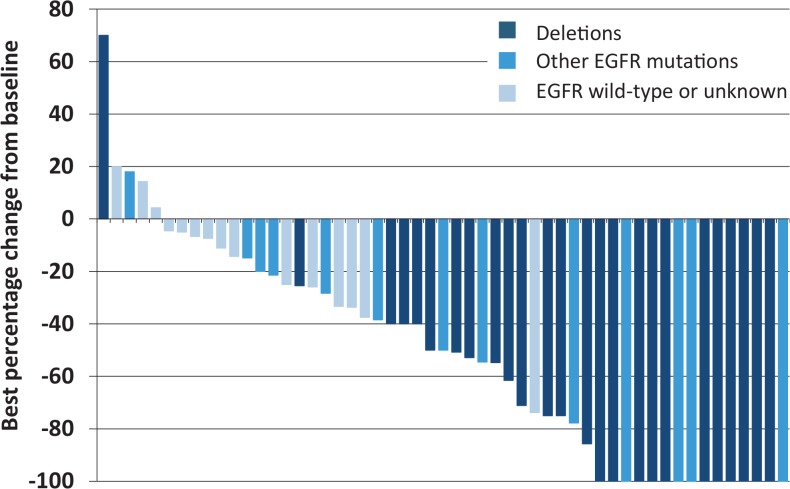
Waterfall plot of best percentage change in tumor size (sum of longest diameters).

Radiologic assessment confirmed CR in 16 (42.1%) and PR in 17 (44.7%), for an overall response of 86.8%. Of the remaining five patients, four patients had minimal response or stable disease, and one had progression. Waterfall plot with the best response is shown in Figure 1.

PET-CT at baseline and after 6 months was performed in 30 patients. Complete remission was documented in 16 patients (53.3%) and PR in 9 patients (40.7%).

**FIGURE 2.**
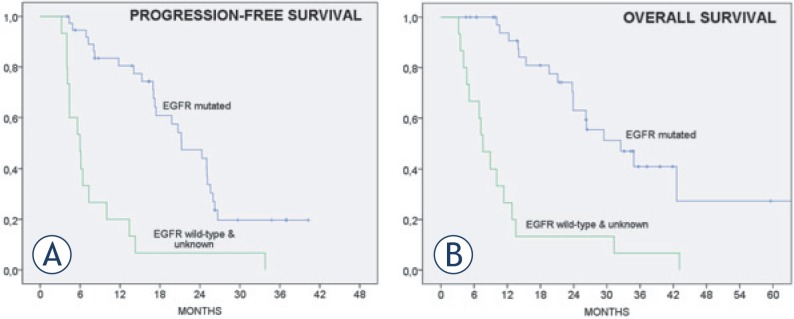
Progression-free and overall survival of treated patients (n = 53). Progression-free survival for epidermal growth-factor receptor (EGFR) wild-type patients (median 6.0 months, 95% confidence interval [CI] 3.9 – 8.1) and for patients with EGFR activating mutations (median 21.2 months, 95% CI 15.3 – 27.1) Overall survival for EGFR wild-type patients (median 7.6 months, 95% CI 5.0 – 10.2) and for patients with EGFR activating mutations (median 32.5 months, 95% CI 21.2 – 43.7)

Median PFS for all EGFR mutant patients was 21.2 months (95% CI 15.3 – 27.1 months) (Figure 2). No significant difference in PFS was seen when comparing patients with exon 19 deletions to those with other mutations (data not shown).

The most frequent sites of progression were bone (10), lung (10), brain (6), liver (3), or distant lymph nodes (3). Two patients with brain metastases and one patient with diffuse progression in the liver did not receive additional systemic treatment. In 17 patients, treatment with erlotinib continued beyond progression. Other choices were gefitinib or afatinib (8 patients) or different combinations of cytotoxic chemotherapy (6 patients); more than one treatment option per patient may apply.

Median survival for patients with EGFR activating mutations was 32.5 months (95% CI 21.2 – 43.7). Patients with initial performance status 0-1 had longer OS, when compared to those with PS 2-3 (34.8 months, 95% CI 22.0 – 47.7 *vs* 21.1 months, 95% CI 9.1 – 33.1; p = 0.08). At the close-out date (April 22, 2014), 20 patients are alive, of whom 10 are still in complete remission and continue with maintenance erlotinib.

## Discussion

The concept of pharmacodynamic separation of cytotoxic drugs and TKIs for patients with advanced NSCLC has been tested in several clinical trials. Six trials enrolled patients in progression after chemotherapy^20^–^24^ or after TKI^25^, with no convincing evidence regarding the advantage of intercalated therapy over conventional choices of second-line therapy. This negative experience is not unexpected: it is reasonable to assume that patients in progression after prior systemic therapy are less likely to respond to a schedule including the category of drugs to which resistance already developed.

Among trials on treatment-naive patients, the closest resemblance to our approach was a US-UK trial of paclitaxel and carboplatin on day 1 and erlotinib on days 2 to 15 of a 3-weekly cycle in the intercalated treatment arm, compared to monotherapy with erlotinib.^26^ Yet, this trial recruited only 15 patients with EGFR activating mutations of whom only 6 were randomised to the intercalated treatment, a figure too small for any meaningful conclusion.

FASTACT^27^ and FASTACT 2^28^ trials demonstrated that erlotinib offers no benefit over chemotherapy alone for EGFR wild-type patients, while those with activating mutations clearly benefit from addition of erlotinib. In hindsight, the design of these two trials was suboptimal for two reasons. First, in the intercalated schedule, timing of erlotinib on days 15 to 28 of a 4-weekly cycle is – in our opinion – questionable. To avoid TKI-induced cell cycle arrest in G1 of the mitotic cycle resulting in putative chemoresistance, »wash-out« period for TKI should be before, rather than after the next application of cytotoxic drugs. The second concern is the choice of chemotherapy alone for the control group. While chemotherapy was indeed the standard treatment for advanced NSCLC some years ago, we now have clear evidence of superiority of TKIs for EGFR mutated patients. Thus, superior survival in the intercalated arm is not unexpected and cannot provide an answer to its potential advantage over treatment with TKI alone.

We would now like to offer comments on our trial with selection of patients, schedule, side effects, response to treatment and future perspectives.

Patients recruited in our trial were all chemo-naive. Factors predicting sensitivity to TKIs were considered in defining the inclusion criteria: smoking status for the first period and EGFR mutations for the second period of recruitment. In other aspects, the population of patients may be considered as prognostically unfavourable, with inclusion of 37% of patients in PS 2-3, 96 % in stage IV, and 74% with 2 or more metastatic sites. In addition, 28% of patients had brain metastases, a frequent metastatic site in EGFR mutated NSCLC.^29^ All our patients were Caucasians. These factors should be considered when comparing the experience to other similar trials.

In the cytotoxic part of our schedule, gemcitabine and cisplatin were applied on days 1, 2 and 4. Such a compressed schedule was chosen in order to gain four more days for erlotinib. According to a report from Hangzhou, China, a similar platin-based doublet with gemcitabine on days 1 and 5 has been found active and well tolerated.^30^ Myelotoxicity after chemotherapy and skin toxicity in the period of maintenance treatment were expected and manageable. Due to 4 cases of grade 4 thrombo-embolic events, routine thromboprophylaxis with low-molecular weight heparin is recommended.

Regarding efficacy, two very distinct groups emerge. Although the number of EGFR wild-type patients was small, it is clear that the objective response rate (33%), median PFS (6.0 months) and median OS (7.6 months) are not superior to the experience with platinum-based doublets alone. On the other hand, the intercalated regimen for EGFR mutated patients is very promising. In a population of patients including those with poor prognostic factors, high proportion of complete or partial responses (42.1% and 44.7%, respectively), long median PFS (21.2 months) and OS (32.5 months) were recorded – figures which are well above most results reported so far for Caucasian patients. According to several clinical studies and to a survey of routine clinical practice, overall response rate to TKIs as monotherapy is around 70% with less than 10% complete remissions, and median PFS is between 9 and 14 months.^19^,^31^

Two explanations are offered for the high efficacy of the intercalated therapy. First, this schedule combines three drugs with proven activity, different mechanisms of action, and different toxicity profiles and at the same time applies the principle of pharmaco-dynamic separation to avoid their mutual antagonistic effect. Second, incorporation of erlotinib into the chemotherapy schedule fills the gaps between individual applications of cytotoxic drugs and thus prevents repopulation of the tumor which may be among the decisive factors for failure of standard chemotherapy schedules for solid tumor.^32^

In conclusions, addition of erlotinib to the doublet of gemcitabine and cisplatin in an intercalated schedule was of no benefit to EGFR wild-type patients. On the other hand, the experience for patients with EGFR mutated advanced NSCLC is very promising. The real value of the concept of intercalated therapy will be established in a randomised trial against monotherapy with a TKI as the current standard of treatment for patients with advanced EGFR mutated NSCLC.

## Figures and Tables

**TABLE 1.** Demographics, prognostic factors, extent of disease and type of EGFR mutations

		**All 53 patients**	**EGFR mutated 38 patients**	**EGFR wild type 15 patients^a^**
**AGE**	median	57	61	45
	range	25 – 74	37 – 74	25 – 73
**GENDER**	male	25	17	8
	female	28	21	7
**SMOKING**	never smoker	33	24	9
	light smoker (< 10 pack years)	11	5	6
	smoker	9	9	0
**PERFORMANCE STATUS**	ECOG PS 0	12	10	2
	1	30	20	10
	2	8	6	2
	3	3	2	1
**STAGE**	III B	2	1	1
	IV	51	37	14
**SITE(S) OF METASTATIC DISEASE**	bone	35	24	11
	distant lung	25	18	7
	pleura and pericardium	24	16	8
	liver and/or suprarenal	17	11	6
	brain (after whole-brain radiotherapy)	15	13 ^b^	2
	distant lymph nodes and/or soft tissues	14	10	4
**NUMBER OF METASTATIC SITES**	1	14	10	4
	2	17	14	3
	3 or more	22	14	8
**TYPE OF EGFR MUTATION**	Exon 19 deletion ^c^	25	25	n. a.
	G719X ^c^	4	4	n. a.
	L858R	9	9	n. a.
	S 768i	1	1	n. a.

EGFR = epidermal growth factor receptor

a Includes 3 patients for whom ERGF status could not be determined

b Includes 1 patient with asymptomatic untreated multiple brain metastases

c One patient had deletions and G719X mutation

**TABLE 2.** Treatment toxicity

		**All 53 patients**	**EGFR mutated 38 patients**	**EGFR wild type 15 patients ^a^**

	**Grade**	**INDUCTION/MAINTENANCE**	**INDUCTION/MAINTENANCE** **^b^**	**INDUCTION/MAINTENANCE ^c^**
**Anemia**	2	14/2	11/2	3/0
	3	1/0	1/0	
**Neutropenia**	2	15/0	12/0	3/0
	3	5/0	4/0	1/0
	4	2/0	2/0	
**Thrombocytopenia**	2	4/0	3/0	1/0
	3	2/0	2/0	
**Nephotoxicity**	2	2/0	1/0	1/0
**Skin toxicity ^d^**	2	11/16	8/11	3/5
	3	4/14	3/13	1/1
**Nausea/vomiting**	2	6/0	4/0	2/0
**Asthenia**	2	2/2	1/2	1/0
**Thrombo-embolic events**	2	1/0	1/0	
	4	4/0	4/0	
**Diarrhea**	2	5/2	3/1	2/1

EGFR = epidermal growth factor receptor

a Includes 3 patients for whom EGFR status could not be determined

b All 38 patients continued with maintenance erlotinib

c 11 patients continued with maintenance erlotinib

d Leading to reduced daily dose of erlotinib to 100 mg (14 patients), 75 mg (6 patients) or 50 mg (5 patients)
